# Vancomycın resıstant enterococcus bacteremıa ın a patıent wıth *Pneumocystis jiroveci* pneumonıa, granulocystıc sarcoma and acute respıratory dıstress syndrome

**DOI:** 10.11604/pamj.2014.17.49.3246

**Published:** 2014-01-23

**Authors:** Julide Celdir Emre, Aysegul Baysak, Adnan Tolga Oz, Gulfem Ece, Bilgin Arda, Feza Bacakoglu

**Affiliations:** 1 Turgutlu State Hospital, Department of Chest Diseases, Manisa, Turkey; 2 Izmir University School of Medicine, Department of Chest Diseases, Izmir, Turkey; 3 Izmir University School of Medicine, Department of Medical Microbiology, Izmir, Turkey; 4 Ege University School of Medicine, Department of Infectious Diseases and Clinical Microbiology, Izmir, Turkey; 5 Ege University School of Medicine, Department of Chest Diseases, Izmir, Turkey

**Keywords:** Granulocystic sarcoma, Pneumocystis jiroveci pneumoniae, vancomycin resistant enterococci, (VRE)

## Abstract

In this case report we aimed to present a patient with granulocytic sarcomaa, neutropenic fever, ARDS and Pneumocystis jirovecii pneumoniae that was hospitalized in our intensive care unit. The patient recovered and then developed vancomycin resistant enterococci (VRE) bacteremia due to port catheter during follow up. The patient had risk factors for VRE bacteremia and he was administered linezolide without removing the catheter. He was discharged with recovery.

## Introduction

Granulocytic sarcoma is a rare extramedullary tumor that originates from granulocytic cells. Granulocytic sarcoma may develop during acute myeloid leukemia, myelodysplastic syndrome and myeloproliferative disorders. It is usually detected on skin,bones, soft tissue and periost layer. It is common in children and adolescents and equally shown in both genders. This is a sign of bad prognosis [1]. Surgical excision, combined chemotherapy, and radiotherapy are treatment models [2]. Allogenic or autologous bone marrow transplantation are additional treatment to systemic chemotherapy in case of coexistence of acute leukemia and granulocytic sarcomae [1]. Recently immunosuppressed cases have increased due to an increment in cytotoxic and corticosteroid treatment (organ transplantation, systemic disorders, etc.) [3, 4]. Neutropenia is defined as neutrophil count less than 500/mm^3^or decreasing under 500/mm^3^ in 24-48 hours. Febril neutropenia, is defined as fever higher than 38.3°C or higher than 38°C during one hour [3]. Pulmonary complications are ictu causes of morbidity and mortality in immunosuppressed cases. In these cases mortality develops depending on underlying disorders, infectious agent and type of complication. Early diagnosis, and treatment are important and multidiciplinary approach is needed. Prematures, children with primary immunodefiency, hematological malignities, organ transplantation, immunosuppresive treatment are under risk of P.jirovecii infection other than AIDS. *Pneumocystis jiroveci* pneumoniae is a clinical icture with diffuse interstitial infiltration. Besides normal pulmonary X-ray, cystic form with increased risk of spontanous pneumothorax, paranchymal consolidation, multiple nodules, pleural fluid, and enlargement of lymph nodes may be other radiological images [5]. Enterococcal infections have increased due to increment in third generation cephalosporin use in 1970s. First isolation of VRE was in 1978 and then globally spreaded and became one of the most important reason of hospital infection. *Enterecoccus feacalis* and *Enterecoccus faecium* are the most common strains [6]. In this case report we aimed to present a patient with granulocytic sarcomae, neutropenic fever, acute respiratory distress syndrome (ARDS) and Pneumocystis jiroveci pneumoniae that was hospitalized in our intensive care unit.

## Patient and observation

**Figure 1:**
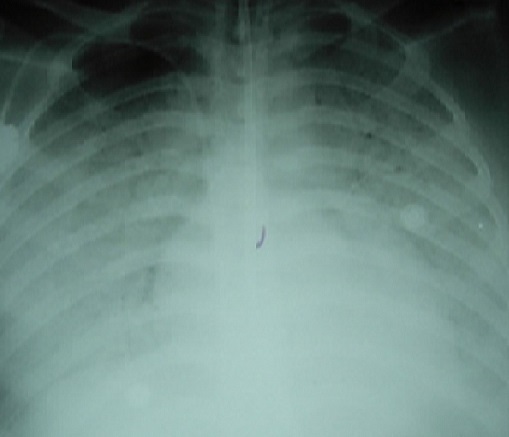
The chest X-ray image revealed bilaterally increased non-homogeneous density on all zones

**Figure 2:**
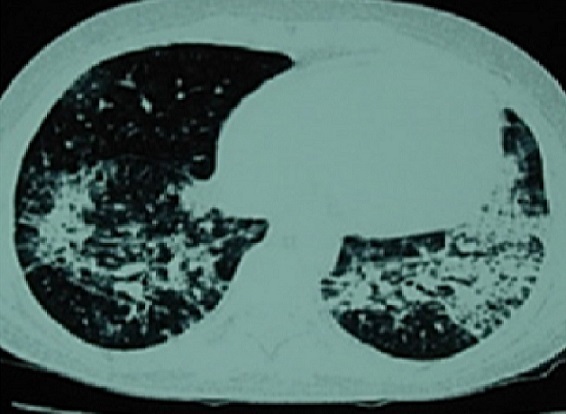
High resolution computerized tomography (HRCT) image of the patient

**Figure 3:**
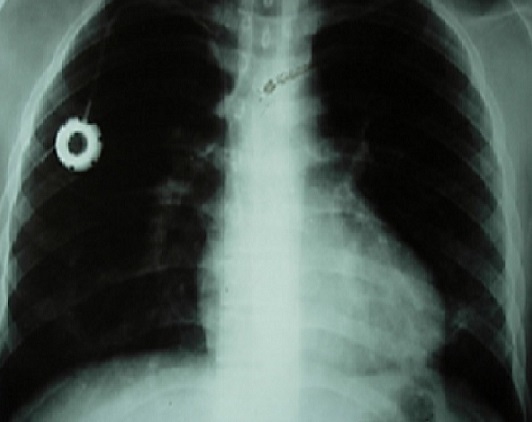
Radiological recovery of the patient on chest X-ray

Nineteen year old male patient had left arm pain two months ago and afterwards he was diagnosed as granulocytic sarcoma. On the seventh day of his chemotherapy neutropenic fever developed. He was administered piperacillin/tazobactam, amikacin; but fever continued and teicoplanin was initiated. Then the antibiotics were stopped and amphothericin B and meropenem was begun. On the twentyfifith day of the chemotherapy regime neutropenic state ended. He developed fever, dyspnea and hypotension on the 27th day. His physical examination showed cyanosis, dyspnea, increased respiratory sound at the upper and middle lung level bilaterally. The arterial blood gas analysis revealed severe hypoxemia (PaO2/FiO2: 82.5) and he was entubated. The patient was transffered to intensive care unit and mechanical ventilation support was applied. The chest X-ray revealed bilaterally increased non-homogeneous density on all zones (Figure 1). High Resolution computerized tomography indicated frost glass appearance (Figure 2). No growth was reported on microbiological cultures. *Pneumocytis jiroveci* pneumoniae was not detected on broncho alveolar lavage sample by Giemsa stain and trimethoprim/sulfamethoxazole (TMP/SMX) 80mg/day was iniated. Clinical and radiological recovery was obtained (Figure 3). Mechanical ventilation support was stopped on the nineth day. Fever increased on twelveth day. Vancomycin resistant *Enterecoccus feacalis* was detected on blood culture taken from the port catheter and evaluated as hospital infection. Strict infection control measures were taken and the patient was administered linezolide. Peripheral and catheter blood cultures were drawn in 48-72 hours before the removal of the port catheter. The patient was discharged on the twentyfourth day with recovery.

## Discussion

*P. Jiroveci* pneumoniae is a clinical picture in individuals with cellular immundeficiency and fever, cough, progressive hypoxemia and dyspnea develops. The clinical status of the patient may be severe. Diffuse interstitial and perihilar infiltration are typical on chest X-ray [7]. Fever and dyspneae may appear a few days earlier. P. *jiroveci*cannot be detected on culture. The diagnosis can be done by direct microscopic examination of the lower respiratory tract samples with Giemsa and Wright stain and PCR. In our case report we could not show P. *jiroveci* on bronchoscopic samples and ampiric treatment was initiated considering hypoxemia and radiological image.

The treatment of *P. jiroveci* pneumoniae requires TMP/SMX (15 mg/kg/day) for 14-21 days as the first line agent. In cases with mild to severe hypoxemia (PaO2 < 70 mmHg) corticosteroid treatment potentiates oxygenation and decreases mortality. The recommended treatment requires 40 mg/day twice for 5 days, 40 mg/day for five days and 20 mg methylprednisolone for 11 days [3]. In our case TMP-SMX and methylprednisolone were iniated and clinical and radiological recovery were obtained. Also dyspnea healed.

Enterococci are a part of the gastrointestinal system and vaginal flora and infections are commonly due to endogeneous flora. The bacterial isolate is transferred to patients by contamianted hands and fomites. Recently there is an increment in nosocomial infections due to enterococci. VRE infections particulary increased in patients with underlying disorders. *E. Faecium* strains show 47% vancomycin resistance [8, 9]. The risk factors for VRE infection are immunsuppression, long duration of hospitalization, and broad spectrum antibiotic. Our patient had all of these risk factors. VRE was reported on port catheter which was used to administer chemotherapy [9]. Linezolid is the only member of synthetic oxyzolidone family and is active in-vitro against resistant Gram positive cocci such as vancomycin resistant *Enterococcus faecalis* and *Enterococcus faecium* and methicilin resistant *Staphylococcus* aureus [9, 10]. The approval was declared in our country in 2005. Our case was treated with linezolide without removing the catheter.

## Conclusion

Chemotherapy and other treatment modalities have increased survey; but long duration of hospitalization, and broad spectrum antibiotic use increase the risk of resistant infections. Appropiate microbiological culture and multidiciplinary approach can decrease mortality.
